# Supplementary material for: New RT-PCR Assay for the Detection of Current and Future SARS-CoV-2 Variants
Source: Viruses. 2023 Jan 11;15(1):206. doi: 10.3390/v15010206 (PMC9863853; doi:10.3390/v15010206)
Supplement: Supplementary file 1 [file viruses-15-00206-s001.zip › viruses-1984386-supplementary.pdf]

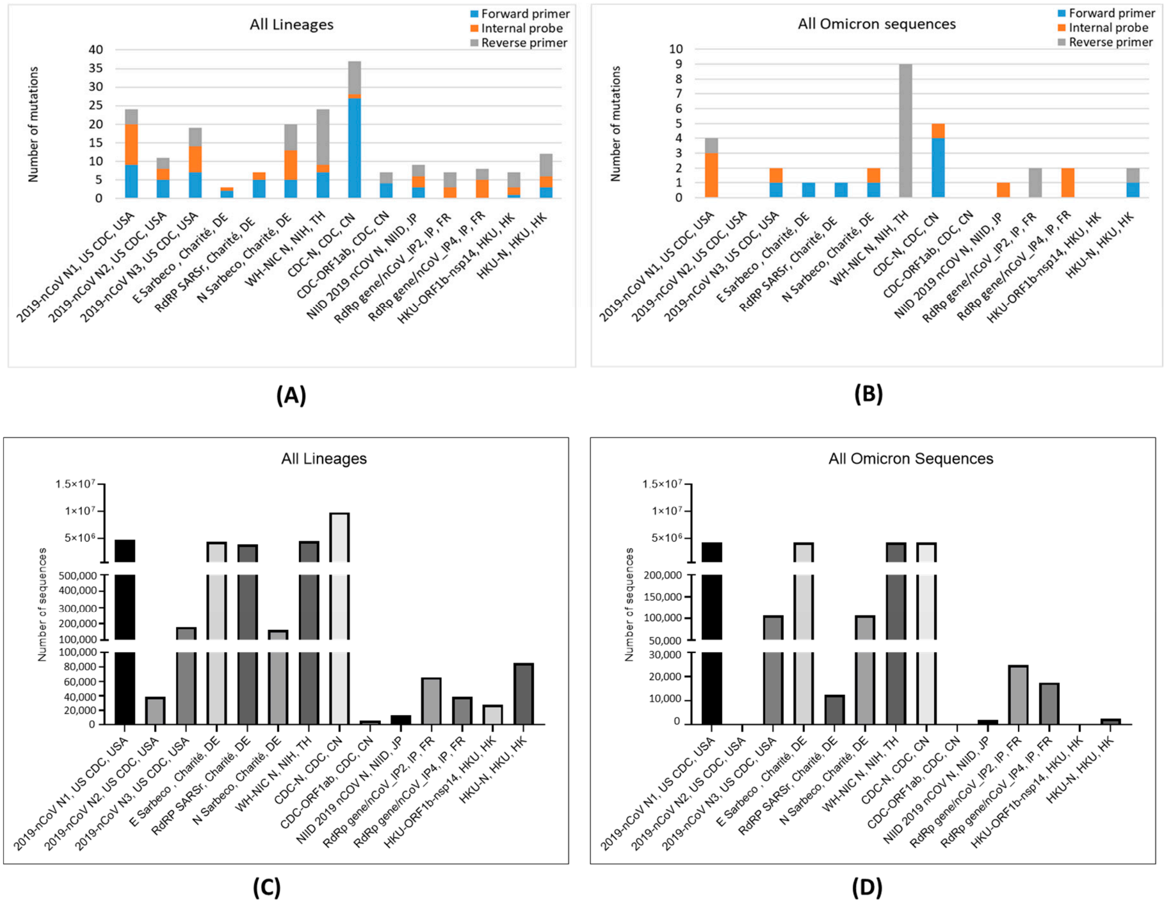

Figure S1. Mutations on targeted regions of primers/probe of WHO-recommended RT-PCR assays. (**A**). Mutations present on all SARS-CoV-2 consensus sequences. Nucleotide alignment between the primers/probes of the 14 WHO-recommended RT-PCR assays (Supplementary Table S1) were performed on the 1815 consensus sequences generated as described in the Materials and Methods section. These sequences are representative of a total of 10,452,789 GISAID sequence submissions. Columns indicate the number of non-redundant mismatches/gaps present in the primers/probe of the various assays. Further information on the alignments that generated the figure are in Supplementary information 1. (**B**). Mutations present on Omicron specific consensus sequences. Alignment was carried out with the 203 Omicron-consensus sequences (see also Supplementary Figure 2) ., C-D. Total number of sequences presenting mismatches. (**C**). Column indicate the total number of SARS-CoV-2 genomes whose sequences were deposed in GISAID presenting mutations in the oligonucleotides consensus sequences of the indicated WHO recommended RT-PCR assays. (**D**). Only Omicron consensus sequence are considered.

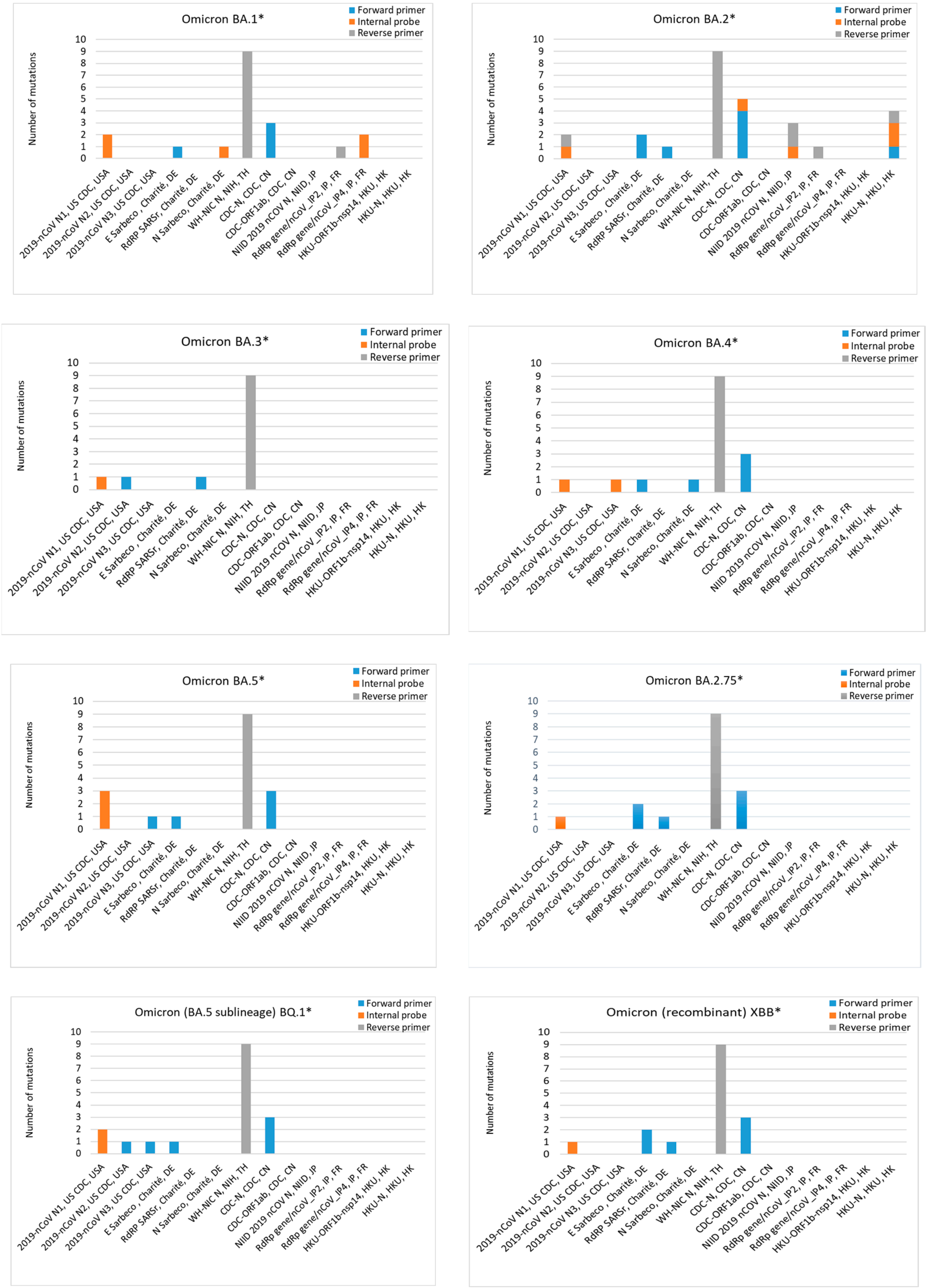

Figure S2. Mutations on Omicron related SARS-CoV-2 consensus sequences leading to mismatches with the oligonucleotides of WHO recommended assays. Alignment was carried out between primers/probe of the various WHO recommended RT-PC assays and the Omicron-consensus sequences representative of the Omicron subvariants indicated.

Developer	Method	All SARS-CoV-2 lineages					
		Perfect		Imperfect		Failure	
		consensus seqs	total seqs	consensus seqs	total seqs	consensus seqs	total seqs
US-CDC, US	2019-nCoV N1	84.35%	60.03%	15.65%	39.97%		
US-CDC, US	2019-nCoV N2	98.07%	99.54%	1.93%	0.46%		
US-CDC, US	2019-nCoV N3	97.80%	99.14%	2.20%	0.86%		
Charité, DE	E Sarbeco	88.43%	63.90%	11.57%	36.10%		
Charité, DE	RdRP SARSr	85.84%	59.81%	14.16%	40.19%		
Charité, DE	N Sarbeco	97.69%	99.19%	2.31%	0.81%		
NIH, TH	WH-NIC N	87.44%	64.04%	1.60%	0.48%	10.96%	35.47%
CDC, CN	CDC-N	37.30%	6.81%	62.64%	93.19%	0.06%	
CDC, CN	CDC-ORF1ab	99.45%	99.96%	0.55%	0.04%		
NIID, JP	NIID 2019 nCOV N	98.90%	99.88%	1.10%	0.12%		
Pasteur Inst, FR	nCoV IP2	99.12%	99.31%	0.88%	0.69%		
Pasteur Inst, FR	RdRp/nCoV IP4	99.28%	99.45%	0.72%	0.55%		
HKU, HK	HKU-ORF1b-nsp14	99.39%	99.73%	0.61%	0.27%		
HKU, HK	HKU-N	97.25%	99.13%	2.75%	0.87%		
JRC-CoV-UCE.1	JRC, EU	99.50%	99.99%	0.50%	0.01%		
JRC-CoV-UCE.2	JRC, EU	99.67%	99.98%	0.33%	0.02%		

Developer	Method	Omicron-related lineages					
		Perfect		Imperfect		Failure	
		consensus seqs	total seqs	consensus seqs	total seqs	consensus seqs	total seqs
US-CDC, US	2019-nCoV N1	0.99%	0.02%	99.01%	99.98%		
US-CDC, US	2019-nCoV N2	100.00%	100.00%				
US-CDC, US	2019-nCoV N3	97.54%	99.04%	2.46%	0.96%		
Charité, DE	E Sarbeco	1.48%	0.01%	98.52%	99.99%		
Charité, DE	RdRP SARSr	99.01%	100.00%	0.99%	0.00%		
Charité, DE	N Sarbeco	97.54%	99.09%	2.46%	0.91%		
NIH, TH	WH-NIC N	5.42%	1.76%	0.00%	0.00%	94.58%	98.24%
CDC, CN	CDC-N	0.49%		99.51%	100.00%		
CDC, CN	CDC-ORF1ab	100.00%	100.00%				
NIID, JP	NIID 2019 nCOV N	99.51%	100.00%	0.49%			
Pasteur Inst, FR	nCoV IP2	98.03%	99.28%	1.97%	0.72%		
Pasteur Inst, FR	RdRp/nCoV IP4	99.01%	99.10%	0.99%	0.90%		
HKU, HK	HKU-ORF1b-nsp14	100.00%	100.00%				
HKU, HK	HKU-N	99.01%	99.98%	0.99%	0.02%		
JRC-CoV-UCE.1	JRC, EU	100.00%	100.00%				
JRC-CoV-UCE.2	JRC, EU	99.51%	99.99%	0.49%	0.01%		

Figure S3. In silico PCR simulations predict potential decay of performance of some of WHO RT-PCR assays. Simulations were performed on 1815 SARS-CoV-2 lineages (upper panel) and on 203 Omicron-related lineages (lower panel). Perfect (green): no mismatches/gaps found in primers/probes targeted regions; imperfect (yellow): up to five mismatches/gaps found in primers/probes targeted regions; failure (red): no amplification is predicted to occur due to a large number of mismatches/gaps found in primers/probes targeted regions.

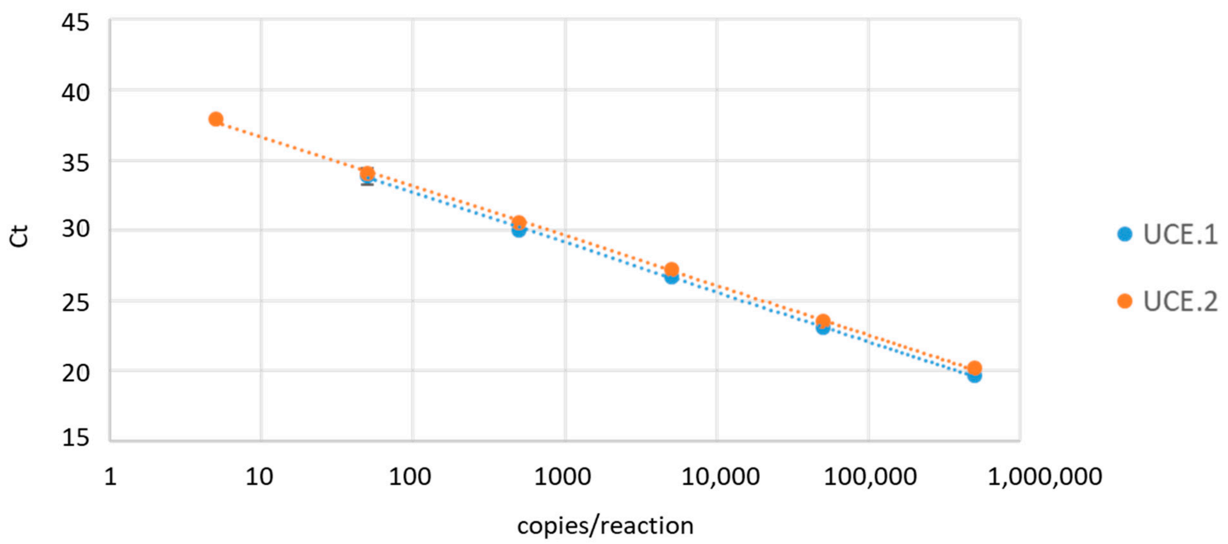

Figure S4. LoD of JRC-CoV-UCE. Serial dilutions of the synthetic Omicron RNA genome at the concentration indicated were analysed with the JRC-CoV-UCE.1 (UCE.1) and JRC-CoV-UCE.2 (UCE.2) methods run as a duplex assay. Each point represents the average of three replicates with relative standard deviation bars.
